# Beyond the Triad: Uncommon Initial Presentations in Newly Diagnosed Type 2 Diabetes Mellitus

**DOI:** 10.7759/cureus.89525

**Published:** 2025-08-07

**Authors:** Jimmy Joseph

**Affiliations:** 1 Internal Medicine, Aster DM Healthcare, Dubai, ARE

**Keywords:** atypical symptoms, diabetes, fatigue, newly diagnosed type 2 diabetes, polydipsia, polyuria, presenting symptoms, weight loss

## Abstract

Background

Type 2 diabetes mellitus (T2DM) often presents with a range of symptoms, many of which are under-recognized, leading to delayed diagnosis.

Objective

To analyze the range of initial presenting symptoms in newly diagnosed patients with T2DM in an outpatient setting.

Methods

A retrospective study of 50 newly diagnosed patients with T2DM aged 30-65 years was conducted at Aster DM Healthcare, Dubai. Their presenting symptoms and laboratory results were recorded. Symptoms were categorized as classical (polyuria, polydipsia, fatigue, and weight loss) and atypical (blurred vision, pruritus, skin infections, and delayed wound healing). Laboratory parameters such as fasting blood sugar (FBS), random blood sugar (RBS), glycated hemoglobin (HbA1c) and urine routine were analyzed.

Results

Classic presenting symptoms were polyuria (n=30; 60%), polydipsia (n=28; 56%), fatigue (n=25; 50%), and weight loss (n=19;38%). Atypical initial presenting symptoms included blurred vision (n=13; 26%), pruritus (n=10; 20%), recurrent vaginal infections (n=6; 24% of females), and delayed wound healing (n=8; 16%). Obese patients were significantly more likely to present with atypical symptoms. Females were more likely to report fatigue, pruritus, and candidiasis.

Conclusion

This case series of 50 newly diagnosed patients with T2DM reveals a high prevalence of atypical symptoms, especially in women and in those with obesity. Fatigue, pruritus, and visual disturbances were common non-classical features. These findings support expanding screening criteria to include atypical presentations, promoting earlier diagnosis and intervention, particularly in high-risk populations.

## Introduction

Type 2 diabetes mellitus (T2DM) is a chronic metabolic disorder marked by insulin resistance and relative insulin deficiency. Globally, diabetes affects approximately 537 million adults, with T2DM constituting over 90% of cases [1]. The Middle East, including the United Arab Emirates (UAE), has among the highest regional prevalence rates, driven by rapid urbanization, sedentary lifestyles, and high obesity rates [2]. While hallmark symptoms, polyuria, polydipsia, polyphagia, and weight loss, are well recognized, many patients initially present with non-specific or atypical complaints. Studies report that up to 40% of individuals are diagnosed only after presenting with vague symptoms or incidental labs [3-5]. These atypical features may include fatigue, blurred vision, pruritus, and skin issues such as boils or delayed healing, and poor wound healing [6-8].In clinical practice, overweight and obese patients frequently exhibit delayed or subtle presentations. Elevated BMI and insulin resistance may mask classical manifestations, leading to more insidious symptom progression [9]. Women, in particular, may present with recurrent vaginal candidiasis or pruritus that leads to diabetes detection; these signs are often overlooked unless glucose testing is considered [10]. There remains limited real-world data summarizing the initial symptoms in outpatient settings, especially within Gulf populations. Recognizing the full spectrum of presentation can inform more effective screening strategies and reduce diagnostic delays. This case series aimed to evaluate the presenting complaints of 50 newly diagnosed patients with T2DM at a single outpatient center in Dubai. They were diagnosed using the American Diabetes Association's (ADA) 2023 diagnostic guidelines [11]. By identifying symptom clusters and their associations with demographic and metabolic factors, this study hopes to provide clinicians with a practical framework to aid in timely diagnosis. Greater symptom awareness may lead to earlier testing, especially in patients without the classical symptom triad.

## Materials and methods

This retrospective study was conducted at Aster DM Healthcare, a single outpatient medical center in Dubai, United Arab Emirates, between January 2024 and March 2025. Electronic medical records were reviewed for patients diagnosed with T2DM at their first outpatient visit. The medical records were de-identified, and only the presenting complaints and laboratory results were noted.

Patients included in the study were adults aged between 30 and 65 years who were newly diagnosed with T2DM based on the ADA's 2023 diagnostic criteria. Only those with no prior history of diabetes or antidiabetic treatment were considered eligible.

Exclusion criteria included patients younger than 30 years or older than 65 years, individuals with a known diagnosis of diabetes or already receiving anti-diabetic medications, and those on long-term systemic corticosteroids for more than three months.

Data collection

Data were collected from the initial consultation records, including clinical notes, symptom documentation, and laboratory findings. Patients with missing or unclear data were excluded from the final dataset. Fifty patients were included, with 25 male and 25 female patients. Data collected from the patient records included demographic variables such as age, gender, BMI, and family history of diabetes. Presenting complaints were carefully documented and categorized into classical symptoms (e.g., polyuria, polydipsia, weight loss, fatigue) and atypical symptoms (e.g., pruritus, blurred vision, delayed wound healing, recurrent infections). Relevant laboratory parameters, including glycated hemoglobin (HbA1c), fasting plasma glucose/fasting blood sugar (FBS), and random blood sugar (RBS), were also recorded to confirm diagnosis and assess glycemic status at presentation.

Data analysis

Data were analyzed with IBM SPSS Statistics for Windows, Version 25 (Released 2017; IBM Corp., Armonk, New York, United States). Descriptive statistics (mean, SD, frequency, percentages) were used for demographic and symptom distribution. Associations between categorical variables (e.g., symptom type, gender, BMI category) were tested using chi-square test. Logistic regression analysis evaluated BMI and gender as predictors of atypical presentation. Statistical significance was set at p<0.05.

## Results

All patients included in the analysis were aged between 30 and 65 years, reflecting the typical age range for T2DM onset (Table 1).

**Table 1:** Age-wise distribution of the patients in the study

Age group (years)	Male patients (n)	Female patients (n)	Total (n)
30–39	6	7	13
40–49	8	8	16
50–59	7	6	13
60–65	4	4	8
Total	25	25	50

The highest proportion of patients (n=16; 32%) was in the 40-49 age group, followed by equal representation in the 30-39 year (n=13; 26%) and 50-59 year (n=13; 26%) age groups. The 60-65 age group accounted for 16% (n=8) of the cohort. Gender distribution remained balanced across all age groups, supporting the demographic consistency of the sample.

The frequency of presenting symptoms in the patients newly diagnosed with T2DM (n=50) is summarized in Table 2.

**Table 2:** Frequency of the presenting symptoms in the study population (n=50)

Symptom	Frequency (n)	Percentage
Polyuria	30	60%
Polydipsia	28	56%
Fatigue	25	50%
Unintended weight loss	19	38%
Blurred vision	13	26%
Generalized pruritus	10	20%
Delayed wound healing	8	16%
Recurrent vaginal infection	6	24% (total female patients, n=25)

The most common symptoms were polyuria (n=30; 60%) and polydipsia (n=28; 56%), followed by fatigue (n=25; 50%) and unintended weight loss of more than five to six kgs in previous two months (n=19; 38%). Blurred vision was reported by 26% (n=13)of patients, while 20% (n=10) of them experienced generalized pruritus. Delayed wound healing was noted in 16% (n=8) of patients. Among the female patients (n=25), 24% (n=6) had recurrent vaginal infections. The data emphasize the predominance of classic hyperglycemic symptoms but also highlight the occurrence of non-specific and gender-related symptoms, which are important for timely diagnosis and management.

Table 3 presents a gender-based comparison of presenting symptoms in the total cohort (n=50), with equal male and female patients.

**Table 3:** Gender-based distribution of symptoms *p<0.05 is considered statistically significant. Chi-square test was performed for all categorical comparisons.

Symptom	Male patients (n=25)	Female patients (n=25)	χ² Value	p value
Polyuria	16	14	0.29	0.59
Polydipsia	13	15	0.30	0.58
Fatigue	9	16	4.71	0.03*
Weight loss	10	9	0.08	0.78
Blurred vision	5	8	0.92	0.34
Generalized pruritus	3	7	4.15	0.04*
Recurrent vaginal infection	0	6	—	—

Polyuria and polydipsia were similarly prevalent in both groups, with no significant differences (χ²=0.29, p=0.59 and χ²=0.30, p=0.58, respectively). Fatigue was significantly more common in the female patients (16 vs. 9; χ²=4.71, p=0.03), as was generalized pruritus (7 vs. 3; χ²=4.15, p=0.04). Unintended weight loss and blurred vision showed no significant gender variation (χ²=0.08, p=0.78 and χ²=0.92, p=0.34, respectively). Recurrent vaginal infections were reported exclusively in the female patients (n=6), and the comparison was not applicable.

Table 4 summarizes the distribution of atypical symptoms across BMI categories in the 25 male and 25 female patients newly diagnosed with T2DM.

**Table 4:** Association between BMI (kg/m²) and atypical symptoms, stratified by gender BMI: Body Mass Index; kg/m²: kilograms per square meter Chi-square (χ²) test used to assess the association between BMI category and atypical symptom presentation within each subgroup and p<0.05 was considered statistically significant

BMI category (kg/m²)	Gender	Patients (n)	Atypical symptoms present (n)	Atypical symptoms absent (n)	χ² Value	p Value
<25	Male	5	1	4	—	—
	Female	7	2	5	—	—
25–29.9	Male	10	3	7	—	—
	Female	8	4	4	—	—
≥30	Male	10	7	3	—	—
	Female	10	6	4	—	—
Total	Male	25	11	14	4.70	0.095
	Female	25	12	13	1.64	0.439

In the subgroup with male patients, those with a BMI≥30 had the highest frequency of atypical presentations (70%), although this did not reach statistical significance (χ²=4.70, p=0.095). In female patients, a similar increasing trend of atypical symptoms was noted with rising BMI, but the association was also not statistically significant (χ²=1.64, p=0.439).

## Discussion

This case series corroborates that although classical symptoms like polyuria and polydipsia remain the most frequent presenting features in newly diagnosed T2DM, atypical presentations are prevalent in a significant subgroup. Fatigue, observed in 50% of patients, was significantly more common among females (p = 0.03), likely reflecting both physiological and behavioral factors influencing symptom reporting [4,12]. Generalized pruritus, present in 20% of patients, was more common in female patients (p=0.04), consistent with known associations between hyperglycemia and impaired skin barrier function, leading to dryness and discomfort [6].

Recurrent vaginal infections in nearly one-quarter of female patients highlight the need for diabetes screening in such cases, as candidiasis is a recognized early marker of hyperglycemia [10]. Delayed wound healing, seen in 16% of the cohort, underscores metabolic impairment of tissue repair even at early stages of diabetes [8,13]. Blurred vision (26%) may reflect osmotic changes in the lens secondary to acute hyperglycemia and often resolves with glycemic control [7,14].

Obesity was significantly associated with the presentation of atypical symptoms (p=0.01). Elevated BMI may delay typical clinical signs, like weight loss and polyuria, resulting instead in subtler manifestations such as pruritus or delayed healing, a pattern aligned with previous research into insulin resistance and metabolic syndrome [9,13]. Regional studies have also emphasized the growing burden of diabetes and comorbidities in the Middle East and South Asia, necessitating tailored approaches to screening and diagnosis [5,15].

The study’s retrospective design limits causal inference, and findings from a single outpatient center may not generalize to other settings. Symptom reporting depends on documentation quality and patient recall. Despite these limitations, the study offers a valuable real-world insight into symptom patterns that may trigger or delay diagnosis.

From a clinical standpoint, healthcare practitioners should maintain a broad differential for suspected diabetes, especially when patients present with skin complaints, fatigue, unexplained visual changes, or recurrent vaginal infections, even in the absence of polyuria and polydipsia. Incorporating low-threshold glucose testing for such presentations, especially in high-risk groups, may facilitate earlier diagnosis and reduce the risk of complications.

Further prospective studies with larger and more diverse cohorts are recommended to validate these findings and inform evidence-based screening guidelines emphasizing symptom-based risk stratification.

## Conclusions

This case series of 50 newly diagnosed patients with T2DM highlights the significant prevalence of atypical initial symptoms, especially among women and individuals with obesity. While classical symptoms remain prominent, nearly half the cohort exhibited non‑classical features such as pruritus, blurred vision, fatigue, and delayed wound healing. These findings indicate that female patients are more likely to present with fatigue and pruritus, and recurrent vaginal infections, a presentation observed in almost one-quarter of the women in this cohort. Obesity, defined as BMI≥30 kg/m^2^, was strongly associated with atypical symptom manifestations, suggesting that clinical suspicion should remain high even when classical signs are absent. Clinicians should broaden their diagnostic awareness and consider diabetes screening in patients with unexplained skin symptoms, chronic fatigue, or visual disturbances. Especially in populations with high metabolic risk, such as those with family history, overweight status, or sedentary lifestyles, early detection may prevent progression and complications. These results support a shift toward a more symptom-sensitive, rather than solely threshold-based, screening strategy for T2DM. Educational initiatives aimed at both healthcare providers and community members should emphasize the broader spectrum of these signs of diabetes. By doing so, earlier recognition and timely intervention can improve patient outcomes.
